# Evaluating HPV Vaccination Behavior and Willingness to Be Vaccinated and Associated Factors Among University Students in Italy

**DOI:** 10.3390/vaccines13040426

**Published:** 2025-04-18

**Authors:** Francesca Licata, Concetta Arianna Scicchitano, Maria Rita Caracciolo, Aida Bianco

**Affiliations:** 1 Department of Health Sciences, School of Medicine, University of Catanzaro “Magna Græcia”, Viale Europa, 88100 Catanzaro, Italy; f.licata@unicz.it (F.L.); mariarita.caracciolo001@stuudenti.unicz.it (M.R.C.); 2 Department of Medical and Surgical Sciences, School of Medicine, University of Catanzaro “Magna Græcia”, Viale Europa, 88100 Catanzaro, Italy; a.scicchitano@unicz.it

**Keywords:** human papillomavirus, Italy, university students, uptake, vaccination, willingness

## Abstract

Objectives: The aim of the present study was to provide insight into potential predictors of HPV vaccination uptake and the willingness to get vaccinated. Methods: This cross-sectional study was conducted among university students using an online, anonymous, self-administered questionnaire. Vaccine hesitancy was measured according to the adult Vaccine Hesitancy Scale (aVHS). Sociodemographic characteristics, beliefs about vaccination decision-making, vaccination behavior, and willingness to receive the HPV vaccine among unvaccinated students, and sources of information about vaccinations were investigated. Results: Among the 542 sampled students, 11.1% were classified as vaccine-hesitant. About one third (31.7%) had not received the HPV vaccination. Males, older students, those who had not received the dTap-IPV booster dose, and those being discouraged from getting vaccinated by a healthcare worker were more likely not to be vaccinated. Students having one parent holding a university degree or higher were more likely to be vaccinated compared to those having parents with a high school diploma or less. Among unvaccinated students, 65.7% were willing to get vaccinated against HPV, and it was positively associated with a low aVHS score and female gender, as well as being enrolled in medical and life sciences majors. Conclusions: Suboptimal HPV vaccination uptake was observed, especially among male and older university students. Insights from the present study highlight the need to address misconceptions about HPV infection and vaccines by providing facts that can be used in conversations with individuals who may feel insecure after having heard various myths about HPV vaccination.

## 1. Introduction

Human papillomavirus (HPV) is the most common sexually transmitted infection that causes growths on the skin or mucous membranes [1]. Moreover, some high-risk HPV strains can lead to cancers [2]. Official epidemiological estimates reported 660,000 cases and 350,000 deaths due to cervical cancer in 2022 worldwide, placing the disease as the fourth most frequently diagnosed cancer and the fourth leading cause of cancer-related death among women [3]. The burden of cancer attributable to HPV among men is estimated to be 70,000 cases, with a higher incidence observed in high-income countries [4]. In Europe, comprehensive epidemiological data on the total number of cancers linked to HPV are lacking. However, regarding cervical cancer, in 2022, Europe recorded 58,219 new cases of cervical cancer, with 2479 of those occurring in Italy. Furthermore, there were 26,950 deaths attributable to cervical cancer across Europe, 1156 of which occurred in Italy [3].

HPV vaccination is a highly effective preventive measure for reducing the HPV burden. The World Health Organization (WHO) Global strategy to accelerate the elimination of cervical cancer advocates the role of prophylactic HPV vaccination as a foundational pillar. It is estimated that the implementation of this strategy could prevent 60 million cervical cancer cases and 45 million related deaths over the next 100 years [5]. According to the WHO, most countries recommend HPV vaccination between the ages of 9 and 14, with the primary target group being females, though vaccination of males and older females, as secondary groups, is recommended where feasible and affordable [6]. In Italy, the quadrivalent HPV vaccine (Gardasil, Merck & Co., Whitehouse Station, NJ, USA) and the bivalent vaccine (Cervarix, GlaxoSmithKline Biologicals, Rixensart, Belgium) have been available free of charge to girls aged 12 years since 2007/2008. In 2014, the U.S. Food and Drug Administration licensed the nonavalent vaccine Gardasil 9 (Merck & Co., Rahway, NJ, USA) with initial approval for use in males and females aged 9–26 years. All HPV vaccines provide protection against HPV types 16 and 18, which cause cancer of the cervix, penis, vulva, vagina, anus, and oropharynx, greatly reducing the likelihood of developing HPV 16- or HPV 18-related cancers [7]. This is important, as virtually all cervical cancers are caused by HPV, with about 70 percent caused by HPV types 16 and/or 18 [8]. More recently, HPV 16 has also been identified as a major cause of oropharyngeal cancers [9], and in the United States of America, the number of HPV-related oropharyngeal cancer cases has surpassed those of HPV-related cervical cancer [10]. Therefore, the Centers for Disease Control and Prevention (CDC) have identified improving the uptake of HPV vaccines as a public health priority to reduce the risk of HPV-related cancers [11].

In Italy, a gender-neutral policy has been implemented since 2015, allowing boys to receive the vaccine as well. Additionally, catch-up vaccinations are offered at no cost for all doses of the HPV vaccine to women up to 26 years old and to men up to 18 years of age who are not fully vaccinated [12].

The National Immunization Prevention Plan provides additional free-of-charge vaccinations for adolescents aged 11 to 18 years, including a booster dose against diphtheria, tetanus, pertussis (dTaP), and poliomyelitis (IPV), which are mandated by law, and vaccines targeting meningococcal serogroups A, C, Y, and W-135 (MenACWY) [12]. The National Immunization Program aims for high coverage rates, with targets set at 95% for HPV and MenACWY vaccinations and ≥90% for the dTap-IPV booster dose by the age of 15 years [12]. During the COVID-19 pandemic, routine adolescent vaccination rates were affected by disruptions in healthcare services and vaccine confidence issues. Data from the Italian Ministry of Health showed that, as of 2023, 69.6% of eligible females and 58.5% of eligible males born in 2008 had received HPV vaccination [13]. It seems clear that national HPV immunization campaigns are facing challenges in achieving the desired high coverage rates.

While it is still best for children to be vaccinated around age 11 or 12, there are still many benefits to getting the vaccine later in life. College presents a valuable opportunity to provide catch-up HPV vaccination to students who were not vaccinated against HPV previously. When teens enter college, many move away from their parents, who may have had roles in influencing their healthcare decisions. Determining the modifiable or influencing factors that lead some students to get vaccinated is pivotal to understanding why young adults choose not to receive the vaccine to develop catch-up vaccination programs that address barriers and increase vaccine uptake.

With this in mind, the present study was conducted in order to (1) assess the uptake of HPV vaccination; (2) evaluate willingness to receive vaccination among unvaccinated individuals; (3) identify potential predictors of uptake and willingness to receive HPV vaccination.

## 2. Materials and Methods

### 2.1. Study Design and Setting

The present cross-sectional study was conducted between 7 March and 3 April 2022 in a sample of students enrolled at the “Magna Græcia” University of Catanzaro, located in the Southern part of Italy. The study reporting followed the Strengthening the Reporting of Observational Studies in Epidemiology (STROBE) guidelines for observational studies [14] (Supplementary Materials).

### 2.2. Study Population and Data Collection

The inclusion criteria to participate in this study were as follows: (i) being aged between 18 and 30 years and (ii) being registered as an undergraduate student at the university. The exclusion criterion was the inability to understand, speak, or read Italian. A cut-off age of 30 years was chosen to focus on young adults.

Approval for data collection was obtained from the university administration. After approval, data were collected anonymously via a self-administered online questionnaire using Google Forms^®^. Based on the eligibility criteria, a sampling frame of students was assembled by the university administration. Then, all the students in the sampling frame were invited to participate in this study and received the survey link through their institutional email. The first page of the online survey included an explanation of the study’s rationale, aims, and how the data would be handled. It stated that eligible participants were not required to complete the questionnaire, that all collected data would remain confidential, and that no identifiers link students to their responses. Only students who selected the “agree” option were directed to fill out the questionnaire. To avoid any multiple submissions, the survey format emailed to the potential participants was limited to one response per person and required respondents to sign in with a Google account, even though email addresses were not collected. This ensured that each participant could complete the questionnaire only once using their personal Google account. No payment or incentives were offered to participants for taking part in this study.

### 2.3. Questionnaire Design

The questionnaire was designed after an extensive review of the literature [15,16,17,18], and it was pretested among a small group of 20 eligible students who were not included in the final sample. Based on the feedback received during this phase, minor adjustments were made to the survey’s layout to facilitate responses from devices other than computers (e.g., smartphones). The final questionnaire took about 10 min to complete and consisted of 31 questions grouped into 4 sections. The first section gathered information about the participants’ sociodemographic characteristics. The second section investigated vaccine hesitancy tested with the adult Vaccine Hesitancy Scale (aVHS) [17,18] and the beliefs related to vaccination decision-making. The aVHS consists of a validated 10-item questionnaire rated on a 5-point Likert scale, with answer choices ranging from “strongly disagree” to “strongly agree”. Each response is assigned a score from 1 to 5, where a score of 1 indicates the least hesitancy and a score of 5 indicates the most hesitancy regarding vaccines. Three of the items are reverse-coded. The total score ranges from 10 to 50, with scores above 24 indicating vaccine hesitancy. The third section investigated adherence to recommended (i.e., HPV) and mandatory (i.e., dTap-IPV) vaccinations during adolescence, and among unvaccinated responders, it also assessed their willingness to receive HPV vaccination and related reasons. The final section examined sources of information about vaccinations, related satisfaction, and the need for additional information on the topic.

Ethical approval of this study was granted by the Calabria Centre Local Human Research Ethics Committee (ID No. 277/15/07/21).

### 2.4. Statistical Analysis

All collected variables were summarized using means and standard deviations (SD) when normally distributed. Medians and interquartile ranges (IQRs) were used in cases of deviations from normality. The skewness of the variables was estimated using the Shapiro–Wilk tests. Categorical variables were expressed as percentages.

Multiple stepwise logistic regression with backward elimination models were developed according to the Hosmer and Lemeshow strategy [19], and independent variables with a *p*-value of 0.25 or less in the univariate analysis were included in the models. Scientifically relevant variables were also included in the models, regardless of the results of the univariate analysis, and the rationale for this approach was to provide as complete control of confounding as possible within the given dataset. The models were built to determine the explanatory variables independently related to dichotomous measures of not having received the HPV vaccine during adolescence (Model 1) and willingness to get vaccinated against HPV among unvaccinated individuals (Model 2). The significance level for the variables entering the models was set at 0.05, and the significance level for removing from the model was set at 0.051. The following independent variables were included in all models: gender (male = 0; female = 1), age (in years, continuous), parents’ education level (both holding a high school diploma or lower = 0, one parent holding a university degree or higher = 1, both parents holding a university degree or higher = 2), the aVHS score (continuous), not having received the dTap-IPV vaccine booster dose during adolescence (no = 0; yes = 1), and having met a healthcare worker (HCW) who discouraged vaccination (no = 0; yes = 1). In Model 1, the variables having at least one chronic illness (no = 0; yes = 1) and belief that individuals ought to make vaccination decisions without any influence from family members and/or friends (no = 0; yes = 1) were also included. Model 2 included the major attended (medical or life science = 0, social science = 1; technology = 2). The goodness of fit of the logistic regressions was ascertained through the Hosmer and Lemeshow test. The area under the Receiver operating characteristic (ROC) curve (AUC) was also investigated to evaluate the discriminative ability of the models. Furthermore, model fit was examined through Pearson standardized residuals, which are defined as standardized distances between observed and expected responses. Observations with standardized Pearson residuals exceeding ±2 may indicate a lack of fit, potential outliers, or influential data points [20]. The statistical significance level was fixed at a *p*-value < 0.05. Adjusted odds ratio (OR) and 95% confidence intervals (CIs) were calculated. Data were analyzed using STATA software, version 18 [21].

## 3. Results

### 3.1. Sociodemographic Characteristics

The study sample consisted of 542 students (response rate of 6.6%) with a mean age of 22.9 years (SD ± 2.9). Of the participants, 9.2% reported having at least one chronic illness. More than half (68.2%) were enrolled in medical or life science majors. Additionally, 17.2% participants reported that one parent held a university degree or higher, and 15.9% reported the same for both parents.

### 3.2. Vaccine Hesitancy and Beliefs About Vaccination Decision-Making

The overall median aVHS score was 18.6 (IQR 15–20), and 11.1% of the students were classified as vaccine-hesitant. Most (81.4%) of the students believed that they do not need vaccines for diseases that are not common anymore. More than one-fourth (27.3%) of the students agreed or were unsure regarding the risk of new vaccines compared to older ones. Less than one-sixth (13.3%) of the respondents were concerned about the serious adverse effects of vaccines. About one-fifth (22.9%) of the students were uncertain or even skeptical about the vaccine information provided by the CDC/Ministry of Health. Conversely, most of the participants agreed that vaccines are important to protect their health (96.6%) and the health of others in the community (96.1%), that getting vaccinated is a good way to protect themselves from diseases (95.6%), and that vaccines are an effective preventative measure to prevent many infectious diseases (94.7%) (Table in the Supplementary Materials).

The vast majority of the sample believed that individuals ought to make vaccination decisions without any influence from family members and/or friends (98.7%) and that the overload of information, sometimes contradictory, can reduce vaccination uptake (93.9%). Regarding confidence in vaccines, 11.1% and 7.8% of the students declared that their friends or their parents are against vaccinations, respectively. Furthermore, 31.4% of the students reported that vaccination was discouraged by an HCW due to a history of allergies (36.5%) or chronic illnesses (21.2%) or due to distrustful attitudes towards vaccination by the HCW (24.7%).

### 3.3. Vaccination Behavior and Willingness to Be Vaccinated

About one-third (31.7%) of students self-reported not having received the HPV vaccination during adolescence, and among unvaccinated individuals, 34.3% disclosed his/her unwillingness to get vaccinated against HPV. The most commonly reported reasons for not getting vaccinated were lack of concern about getting infected (42.4%), low perceived benefits of vaccination (25.4%), barriers to accessing vaccination centers (e.g., long waiting list) (13.6%), and lack of recommendation by HCWs (5.1%). Conversely, the most cited reasons for being vaccinated were preventing HPV-related cancers (55.7%), following an HCW’s recommendation (53.8%), preventing HPV infection (47.8%), and adhering to public health authorities’ recommendations (45.1%). Among unvaccinated individuals who were willing to get vaccinated, the most commonly reported reasons were to prevent HPV infection (58.4%) and HPV-related cancer (53.1%), to adhere to public health authorities’ recommendations (49.6%), and following an HCW’s recommendation (23%).

Only 10.5% of the students in the sample declared not having received the dTap-IPV vaccine booster dose.

### 3.4. Logistic Regression Models of HPV Behavior and Willingness to Be Vaccinated

Model 1 in Table 1 identified gender and age as the strongest predictors of not having received the HPV vaccination. Indeed, the odds of not having received the HPV vaccination were significantly lower among female students (OR: 0.08; 95% CI: 0.05–0.13) compared to their male counterparts. Conversely, for each additional year of age (OR: 1.31; 95% CI: 1.21–1.42), the odds of not having received the HPV vaccination increased by 31%. Other significant predictors of not having received the HPV vaccination were not getting the dTap-IPV booster dose during adolescence (OR: 2.47; 95% CI: 1.26–4.84) and being discouraged from getting vaccinated by HCWs (OR: 2.16; 95% CI: 1.36–3.44). Students that had at least one parent with a university degree or higher (OR: 0.46; 95% CI: 0.25–0.86) were less likely to have not received HPV vaccination compared to those whose parents had a high school diploma or less. Model 1 provided satisfactory goodness of fit (*p*-values of 0.17), and the AUC of the model was 0.83, indicating good discriminative ability. The residuals of the model fell within the acceptable range of ±2, suggesting that it adequately captures the structure of the data.

Among unvaccinated individuals, Model 2 in Table 1 revealed that female students were more willing to get vaccinated (OR: 2.30; 95% CI: 1.14–4.64). Similarly, a low aVHS score (OR: 0.91; 95% CI: 0.86–0.97) was a predictor of willingness to receive the HPV vaccination. Otherwise, being enrolled in technology majors (OR: 0.27; 95% CI: 0.08–0.92) compared to being enrolled in medical and life sciences majors and having both parents holding a university degree or higher (OR: 0.35; 95% CI: 0.16–0.79) compared to having both parents holding a high school diploma or lower, were negatively associated with the willingness to receive the HPV vaccination. The goodness of fit of the model was satisfactory (*p*-values of 0.45), and the AUC values (0.70) indicated sufficient discriminative ability of the model. The residuals of the model fell within the acceptable range of ±2, suggesting that it adequately captures the structure of the data.

### 3.5. Sources of Information

Regarding the preferred sources of information about vaccination, almost all students mentioned HCWs (99.5%), followed by friends and/or family members (91.3%) and the Internet (88.6%). Furthermore, students reported being most satisfied with the information provided by HCWs (99.5%) and friends and/or family members (42.4%). Almost half (45.6%) of the respondents expressed the need for further information about vaccinations.

## 4. Discussion

The findings of this study provided a thorough insight into the factors and barriers related to HPV vaccination, as well as circumstances associated with the willingness to get vaccinated among unvaccinated young adults. These findings could be useful for designing effective policies and strategies to improve HPV vaccination rates and, ultimately, decrease the burden of HPV-related diseases.

One of the key findings of the present study is the suboptimal HPV vaccination uptake among the enrolled individuals (i.e., 68.3%), which is far away from the target set by the Italian Ministry of Health (i.e., 95%). This finding is also far from the target set by the WHO, which is one of the three key goals for consigning cervical cancer to history [22]. Indeed, the global strategy of the WHO’s Cervical Cancer Elimination Initiative includes the target of 90% of girls fully vaccinated against HPV by age of 15 years [23]. Hence, the suboptimal HPV vaccination uptake is of concern since it is hindering the goal of elimination of cervical cancer.

Similar to previous studies [24,25,26,27], the strongest predictor of not having received the HPV vaccination is male gender. It could be argued that the lower vaccine uptake among males compared to females may be the result of the initial restriction of national vaccination recommendations to females [28]. In the year 2013, Australia became the first country to introduce a publicly funded national HPV vaccination program, delivering three doses of the quadrivalent vaccine to girls and boys aged 12–13 years [29]. Australia has the highest coverage rate in the world among boys, with 78%, 75%, and 67% coverage for the first, second, and third doses at age 15 years, respectively. Coverage is even higher (86%, 83%, and 78%) in females. The high vaccination coverage can be attributed to school-based delivery of the immunization program and high community acceptance of HPV vaccination as a cancer prevention strategy [29]. In Europe, 40 out of 54 countries across the WHO European region have national HPV vaccination programs for girls. Boys are currently included in national HPV vaccination programs in 18 countries of the WHO European region. A total of 26 countries are therefore currently or will be, including boys in their national HPV vaccination programs, representing almost half (48%) of all countries in the region [30]. The Italian government was the first among the G8 countries to offer the HPV vaccination to both genders in the year 2015. Despite this, the prior restricted vaccination strategy may have contributed to lower familiarity with male vaccination and, ultimately, lower coverage rates among males compared to females. The study’s finding that male gender is the strongest predictor of not having received the HPV vaccination calls for immediate action from public health authorities to clarify the role of HPV vaccination among males, especially in preventing virus transmission to female sexual partners and reaching the target of eradicating cervical cancer, as well as in reducing the burden of HPV-related cancers in men, chiefly oropharyngeal cancers.

HPV vaccination behavior was also strongly predicted by older age. Similar to what occurred in the case of male vaccination, it could be argued that older cohorts, who faced the initial vaccine rollout, struggled with unfamiliarity and safety concerns surrounding a new vaccine [31,32,33]. It is well known that, despite evidence proving vaccine safety, as new vaccines are developed and offered, concerns regarding their safety arise [34,35]. The unbalanced risk perception related to vaccination represents one of the main drivers of vaccination refusal or delay [36,37], especially among parents [38]. Indeed, vaccination refusal is a phenomenon observed especially when making decisions about children’s [39] and adolescent vaccination [40]. Presumably, younger cohorts may have benefited from increased awareness and trust in the vaccine’s safety profiles due to the longer market presence.

Not having received the dTap-IPV booster dose was another significant predictor of HPV vaccination behavior, almost doubling the odds of not being vaccinated against HPV. The uptake of the dTap-IPV booster dose (89.5%) proximates the National Immunization Prevention Plan target (≥90%). Moreover, national official data demonstrate that dTaP-IPV booster dose coverage was unaffected by disruption due to the COVID-19 pandemic [41], in contrast to HPV vaccination coverage. Therefore, the former finding suggests the potential of the mandatory policy of certain vaccinations (i.e., dTaP-IPV booster dose) to positively influence the uptake of recommended vaccinations (i.e., HPV vaccination) [42].

The role of HCWs in vaccination decision-making was confirmed by the study results since students who were discouraged from getting vaccinated by an HCW were significantly more likely to not have received the HPV vaccination. Indeed, the presence or absence of recommendations from HCWs to get vaccinated is a game-changing factor in vaccination decision-making for oneself and/or one’s son/daughter [36,43,44]; this figure was also corroborated by one of the most frequently reported reasons for getting vaccinated (i.e., adherence to HCWs’ recommendation). HCWs play a pivotal role in vaccination decision-making and need adequate training and continuous education aiming at building skills for increasing vaccine acceptance and addressing the questions and concerns of vaccine recipients. Indeed, inconsistent communication from HCWs regarding HPV vaccination, such as presenting the vaccine as optional or information deficits, can undermine the perception of its importance [45,46].

Another key finding of the present study pertains to the proportion of students who are willing to receive the HPV vaccination, i.e., more than half of the unvaccinated students. There is growing evidence that earlier HPV vaccination is better [47], but vaccination at some point is better than none at all [48,49]. Therefore, implementing tailored catch-up HPV vaccination campaigns could enhance uptake among individuals who are not fully vaccinated. All of that being said, public health authorities should consider designing on-campus catch-up vaccination campaigns since the barriers to accessing vaccination centers are one of the reasons why students are not getting vaccinated. In addition, students of medical and life sciences majors were more inclined to get vaccinated, in line with a previous study [26]. Presumably, they are more educated on the topic than students enrolled in different majors. Indeed, previous studies among university students highlighted that those enrolled in medical and life science majors are usually more knowledgeable about specific health matters [50,51] and HPV infection and vaccination [27,52,53]. Therefore, it might be interesting to consider medical and life science students as “champions” actively working to promote HPV vaccination among their peers enrolled in different majors.

As reported in previous studies [54,55], an important predictor of willingness to receive the HPV vaccination was lower vaccine hesitancy. It is widely known that vaccine hesitancy is often driven by misinformation [56,57]. In the present study, the Internet is one of the most common sources of information used by university students. Since online platforms appear to be playing an important role in the spread of misinformation about vaccines, and the university student population could be especially vulnerable to the influence of misinformation via the Internet and social media [58,59], health organizations should not overlook the importance of these channels within their communication strategy, particularly among young individuals who are the most digitally savvy population group. Furthermore, it is noteworthy that the vast majority of the sample cited friends and/or family members as sources of information. The use of informal sources could foster uncertainty compared to formal sources of information, such as HCWs or health organizations. In addition, the students’ view that an overload of information, sometimes contradictory, could reduce vaccination uptake, coupled with the desire for further information on vaccinations exhibited by half of the sample, underscores the urgent need to enhance the accessibility to official health communication channels.

In the present study, it seems clear that students did not perceive themselves as being at high risk for HPV since the most reported reasons for not getting vaccinated were low levels of perceived HPV infection risk coupled with low perceived benefits of vaccination. The observation is of concern since HPV infection is the most common sexually transmitted infection worldwide [2], and it is especially prevalent among young individuals [60,61]. The lack of education regarding sexually transmitted infections, including HPV infection, represents a possible explanation, as suggested by a previous study conducted among parents of male adolescents in the same area [31]. Furthermore, it is alarming that some students did not perceive the benefits of HPV vaccination since more than 15 years of monitoring and research have accumulated reassuring evidence that HPV vaccination provides safe, effective, and long-lasting protection against cancers caused by HPV infections. Addressing these misconceptions by providing facts that can be used in discussions with individuals who may feel insecure after having heard various myths about HPV vaccination is crucial. HCWs should have easy access to key findings that refute these myths to improve communication within the clinical practice to increase vaccine confidence and the likelihood of successful HPV catch-up campaigns.

### Limitations

The potential methodological limitations of this study should be considered when interpreting the findings. First, the cross-sectional design of this study does not allow to conclude causality about the observed associations, and the findings could be viewed as scantly informative for causal inference. Nevertheless, the results provide valuable information on HPV vaccination uptake and willingness that should not be dismissed. Moreover, information from cross-sectional studies is pivotal in providing baseline data to generate hypotheses for further research, which could inform the development of targeted interventions. Second, recall bias was also a risk, especially when the students were asked about vaccination behavior that occurred several years ago, and it could lead to an underestimation of the real uptake. However, we believe recall bias is partly restricted, as it was recommended to the students to look at their official health booklet before answering the questionnaire. Third, the frequency of willingness to get vaccinated against HPV and vaccine hesitancy may not be accurate due to social desirability bias. However, this risk was limited by guaranteeing participants that their responses would be anonymous and could not be traced back to them. Fourth, the low response rate (6.6% in the present study) is an indication of the potential for nonresponse bias. The extent of nonresponse bias depends on the nonresponse rate. However, a high level of nonresponse alone does not necessarily lead to research bias, as nonresponse can also result from a random error (e.g., if nonrespondents missed the email). Nonresponse bias may be low even when the response rate is low, and a recent study found that estimates based on data remained reliable even with a 5–10% response rate with a sample size of at least 500 [62].

## 5. Conclusions

The study findings highlight suboptimal uptake of the HPV vaccination, particularly among male and older university students. Public health authorities should pay considerable attention to formal (i.e., HCWs) and informal (i.e., family members and friends) sources of information that are more frequently used by this population group. Insights from the present study highlight the need to address misconceptions about HPV infection and vaccines by providing facts that can be used in discussions with individuals who may feel insecure after having heard various myths about HPV vaccination.

## Figures and Tables

**Table 1:** Results of the regression models for the potential determinants of the outcomes of interest.

Variables	OR	95% CI	*p*-Value
**Model 1. Outcome: Not having received HPV vaccination during adolescence** *log likelihood = −251.1184 Prob > chi2 < 0.0000; Obs = 542*			
**Age in years, continuous**	1.31	1.21–1.42	<0.001
**Gender**			
Male *	1.00		
Female	0.08	0.05–0.13	<0.001
**Having met an HCW who discouraged vaccination**			
No *	1.00		
Yes	2.16	1.36–3.44	0.001
**Not having received the dTap-IPV vaccine booster dose**			
No *	1.00		
Yes	2.47	1.26–4.84	0.008
**Parents’ education level**			
Both holding a high school diploma or lower	1.00		
One parent holding a university degree or higher	0.46	0.25–0.86	0.015
Both holding a university degree or higher	Backward elimination		
**Adult vaccine hesitancy scale, continuous**	Backward elimination		
**Having at least one chronic illness**			
No *	1.00		
Yes	Backward elimination		
**Belief that individuals make vaccination decisions without any** **influence from family members and/or friends**			
No *	1.00		
Yes	Backward elimination		
**Model 2. Outcome: willingness to receive the HPV vaccination, among unvaccinated students** *log likelihood = −100.08172; Prob > chi2 = 0.0003; Obs = 172*			
**Adult vaccine hesitancy scale, continuous**	0.91	0.86–0.97	0.005
**Parents’ education level**			
Both holding a high school diploma or lower	1.00		
One parent holding a university degree or higher	Backward elimination		
Both holding a university degree or higher	0.35	0.16–0.79	0.010
**Gender**			
Male *	1.00		
Female	2.30	1.14–4.64	0.019
**Major**			
Medical or life sciences *	1.00		
Social sciences	Backward elimination		
Technology	0.27	0.08–0.92	0.036
**Age in years, continuous**	Backward elimination		
**Not having received the dTap-IPV vaccine booster dose**			
No *	1.00		
Yes	Backward elimination		
**Having met an HCW who discouraged vaccination**			
No *	1.00		
Yes	Backward elimination		

* Reference category. 95% CI: 95% Confidence Interval; dTap-IPV: Diphtheria, Tetanus, acellular Pertussis and Inactivated Poliovirus Vaccine; HCW: Healthcare Worker; HPV: Human papillomavirus; OR: Odds Ratio.

## Data Availability

The original data presented in this study are openly available in the Mendeley Data repository (https://doi.org/10.17632/wc5h8mzygr.1).

## Supplementary Materials

The following supporting information can be downloaded at https://www.mdpi.com/article/10.3390/vaccines13040426/s1, Supplementary Material S1: STROBE checklist for cross-sectional studies, Supplementary Material S2: Table: Distribution of responses to the statements of the adult Vaccine Hesitancy Scale (aVHS).

## Abbreviations

The following abbreviations are used in this manuscript:

aVHS	adult Vaccine Hesitancy Scale
CDC	Centers for Disease Control and Prevention
CI	Confidence Interval
dTap-IPV	Diphtheria, Tetanus, acellular Pertussis and Inactivated Poliovirus Vaccine
HCWs	Healthcare Workers
HPV	Human papillomavirus
IQR	Interquartile Ranges
OR	Odds Ratio
SD	Standard Deviation
WHO	World Health Organization
