# Supplementary material for: Evaluating HPV Vaccination Behavior and Willingness to Be Vaccinated and Associated Factors Among University Students in Italy
Source: Vaccines (Basel). 2025 Apr 18;13(4):426. doi: 10.3390/vaccines13040426 (PMC12031029; doi:10.3390/vaccines13040426)
Supplement: Supplementary file 1 [file vaccines-13-00426-s001.zip › vaccines-3543032-supplementary.pdf]

Supplementary Material S1: STROBE Statement—Checklist of items that should be included in reports of *cross-sectional studies*

	Item No	Recommendation	Page(s)
**Title and abstract**	1	(*a*) Indicate the study's design with a commonly used term in the title or the abstract	1
		(*b*) Provide in the abstract an informative and balanced summary of what was done and what was found	1
**Introduction**			
Background/rationale	2	Explain the scientific background and rationale for the investigation being reported	1-2
Objectives	3	State specific objectives, including any prespecified hypotheses	2
**Methods**			
Study design	4	Present key elements of study design early in the paper	2-3
Setting	5	Describe the setting, locations, and relevant dates, including periods of recruitment, exposure, follow-up, and data collection	2-3
Participants	6	(*a*) Give the eligibility criteria, and the sources and methods of selection of participants	3
Variables	7	Clearly define all outcomes, exposures, predictors, potential confounders, and effect modifiers. Give diagnostic criteria, if applicable	4
Data sources/ measurement	8*	For each variable of interest, give sources of data and details of methods of assessment (measurement). Describe comparability of assessment methods if there is more than one group	3
Bias	9	Describe any efforts to address potential sources of bias	9-10
Study size	10	Explain how the study size was arrived at	NA
Quantitative variables	11	Explain how quantitative variables were handled in the analyses. If applicable, describe which groupings were chosen and why	NA
Statistical methods	12	(*a*) Describe all statistical methods, including those used to control for confounding	4
		(*b*) Describe any methods used to examine subgroups and interactions	NA
		(*c*) Explain how missing data were addressed	NA
		(*d*) If applicable, describe analytical methods taking account of sampling strategy	NA
		(*e*) Describe any sensitivity analyses	NA
**Results**			
Participants	13*	(a) Report numbers of individuals at each stage of study—eg numbers potentially eligible, examined for eligibility, confirmed eligible, included in the study, completing follow-up, and analysed	NA
		(b) Give reasons for non-participation at each stage	NA
		(c) Consider use of a flow diagram	NA
Descriptive data	14*	(a) Give characteristics of study participants (eg demographic, clinical, social) and information on exposures and potential confounders	4
		(b) Indicate number of participants with missing data for each variable of interest	NA
Outcome data	15*	Report numbers of outcome events or summary measures	4-5
Main results	16	(*a*) Give unadjusted estimates and, if applicable, confounder-adjusted estimates and their precision (eg, 95% confidence interval). Make clear which confounders were adjusted for and why they were included	5-7
		(*b*) Report category boundaries when continuous variables were categorized	NA
		(*c*) If relevant, consider translating estimates of relative risk into absolute risk for a meaningful time period	NA

Other analyses	17	Report other analyses done—eg analyses of subgroups and interactions, and sensitivity analyses	5,7
**Discussion**			
Key results	18	Summarise key results with reference to study objectives	7
Limitations	19	Discuss limitations of the study, taking into account sources of potential bias or imprecision. Discuss both direction and magnitude of any potential bias	9-10
Interpretation	20	Give a cautious overall interpretation of results considering objectives, limitations, multiplicity of analyses, results from similar studies, and other relevant evidence	7-9
Generalisability	21	Discuss the generalisability (external validity) of the study results	9-10
**Other information**			
Funding	22	Give the source of funding and the role of the funders for the present study and, if applicable, for the original study on which the present article is based	NA

*Give information separately for exposed and unexposed groups.

Supplementary Material S2: Table. Distribution of responses to the statements of the adult Vaccine Hesitancy Scale (aVHS).

Statements (542 respondents)	Strongly disagree		Disagree		Uncertain		Agree		Strongly agree	
	N	%	N	%	N	%	N	%	N	%
Vaccines are important for my health	1	0.2	6	1.1	13	2.4	70	12.9	452	83.4
Vaccines are effective	1	0.2	8	1.5	20	3.7	124	22.9	389	71.8
Being vaccinated is important for the health of others in my community	3	0.6	4	0.7	14	2.6	75	13.8	446	82.3
All routine vaccinations recommended by the CDC/ Ministry of Health are beneficial	5	0.9	8	1.5	42	7.8	108	19.9	379	69.9
New vaccines carry more risks than older vaccines	251	46.3	143	26.4	104	19.2	25	4.6	19	3.5
The information I receive about vaccines from the CDC/ Ministry of Health is reliable and trustworthy	12	2.2	34	6.3	78	14.4	208	38.4	210	38.8
Getting vaccines is a good way to protect me from disease	2	0.4	8	1.5	14	2.6	96	17.7	422	77.9
Generally, I do what my doctor or healthcare provider recommends about vaccines for me	13	2.4	23	4.2	45	8.3	182	33.6	279	51.5
I am concerned about serious adverse effects of vaccines	195	36	166	30.6	109	20.1	50	9.2	22	4.1
I do not need vaccines for diseases that are not common anymore	24	4.4	21	3.9	56	10.3	102	18.8	339	62.6
